# Familiarity, attitude and practice of postgraduate health science students of Pakistan regarding the implication of artificial intelligence in research: an analytical survey

**DOI:** 10.1186/s12909-026-08632-x

**Published:** 2026-01-30

**Authors:** Najia Rahim, Shagufta Nesar, Unaiza Pervaiz Hashmi, Abdur Rasheed, Sania Majeed, Bushra Noor, Ale Zehra, Mahmooda Naqvi, Muhammad Salahuddin Usmani, Shazia Jamshed

**Affiliations:** 1 Department of Pharmacy Practice, Faculty of Pharmaceutical Sciences, Dow University of Health Sciences, Karachi, Pakistan; 2 Jinnah College of Pharmacy, Sohail University, Karachi, Pakistan; 3 Jinnah Medical and Dental College, Sohail University, Karachi, Pakistan; 4 School of Public Health, Dow University of Health Sciences, Karachi, Pakistan; 5 International Center of Chemical and Biological Sciences, University of Karachi, Karachi, Pakistan; 6 Pakistan Institute of Living and Learning, Karachi, Pakistan; 7 Sindh Medical College, Jinnah Sindh Medical University, Karachi, Pakistan; 8 Department of Pharmacy Practice, Faculty of Pharmacy, Jinnah University for Women, Karachi, Pakistan; 9 Department of Pharmacy Practice, Shifa College of Pharmaceutical Sciences, Shifa Tameer-e-Millat University, Islamabad, Pakistan; 10 Department of Pharmacy Practice, School of Pharmacy, International Medical University, Kuala Lumpur, Malaysia

**Keywords:** Artificial intelligence, Pakistani postgraduate health science students, Attitudes, Practice

## Abstract

**Background:**

Artificial intelligence (AI) is one of the areas of healthcare research that is now growing at the fastest rate in the healthcare sector. This is momentous to know about Pakistani postgraduate health science (PGHS) students’ familiarity, attitudes, and practice regarding the implication of AI in research. Therefore, the current study was designed and conducted to explore PGHS students’ opinions.

**Method:**

A cross-sectional, analytical survey was used. A questionnaire was adopted and validated to seek the opinion of PGHS students. The test-retest method was used to measure reliability. Exploratory factor analysis was used to assess the construct validity of the attitude and practice scales in the questionnaire. Moreover, confirmatory factor analysis was also performed. Non-parametric Fisher Exact test was used to find any significant associations between the items and students’ characteristics.

**Results:**

A pre-test (*n* = 20 students) was conducted to assess the questionnaire’s suitability and readability, and no feedback indicated a need for modifications. The test-retest method was employed, and the total scores were then correlated, yielding an intraclass correlation value of 0.924. Factor analysis of the scale revealed a single common factor with all loadings exceeding 0.40. Cronbach’s alpha was found in the range of 0.811–0.866. A total of 257 PGHS students responded to the questionnaire. A significant majority (64%) reported no formal training in AI. The majority of students (76%) planned to integrate AI in the future. Most students agreed or strongly agreed on the usefulness of AI in academic research (93%). Almost 60% of students strongly agreed or agreed that AI will transform health care and clinical research. Half of them believed that AI tools would not replace researchers.

**Conclusion:**

PGHS students have a positive opinion regarding AI and acknowledge its potential to improve problem-solving and research. However, there are serious worries about the ethical ramifications of AI, accuracy, and employment displacement. The majority of students supported the introduction of AI training, which seems to be a current deficiency in the health science curriculum in Pakistan. To promote the broader use of AI-enabled healthcare research, a multifaceted strategy that addresses specific issues, provides customized training, and includes education is required.

**Supplementary Information:**

The online version contains supplementary material available at 10.1186/s12909-026-08632-x.

## Introduction

Artificial intelligence (AI) imitates human problem-solving and decision-making skills through the application of computers and other technologies. The advent of AI represents a modern age that has the potential to completely transform our everyday workspace. Similar to many other sectors, AI has begun to infringe on the healthcare profession and is currently impacting it both academically and practically [1]. AI is modifying the healthcare sector with the potential to completely change diagnosis, treatment, and decision-making [2]. AI tools like machine learning and natural language processing have proven to be very proficient in decoding complex clinical data, finding trends, and making tailored suggestions [3]. However, patient privacy, ethical concerns, input data dependability, and adversarial assaults are some of the issues that need to be resolved [4]. Therefore, before utilizing AI in research and practice, it is equally crucial to recognize its challenges and limitations [5].

Health education still lags behind the advancements of AI, despite the growing interest in new discoveries [6]. The introduction of AI training into undergraduate health science education has been sluggish, despite numerous calls to action [7]. Similar is the case of postgraduate education. As a result, investigating the perspectives and existing knowledge of health science students can be a useful tool for identifying problem areas and a crucial resource for directing the inclusion of AI in health education.

Postgraduate research is evolving because of AI-driven technologies, including automated data analysis, academic writing assistance, and machine learning models [8]. Postgraduate students typically display a wide range of perspectives about AI because of their exposure to AI applications, research interests, and academic backgrounds [9]. The reason is that AI is an effective tool that improves research productivity, automates tedious activities, and yields fresh insights in a variety of fields related to healthcare [10]. However, students applaud these developments; some are concerned that an excessive dependence on AI may erode research creativity and critical thinking [11]. Although they acknowledge its potential, they emphasized the necessity of regulation and ethical considerations when implementing it in research and practice [12].

PGHS students’ familiarity, attitude, and perceptions of AI are dynamic and change according to their context. Establishing effective postgraduate education and research efforts that raise students’ interest in using AI requires an understanding of the interaction between familiarity, attitudes, and perceptions. Little research has been done on these variables among postgraduate students, particularly health science students. Although researchers conducted study on the knowledge, attitudes, and practices of postgraduate health science (PGHS) students regarding AI [13]. Compared to their undergraduate colleagues, postgraduate students may have distinct degrees of knowledge and viewpoints due to their more advanced training and frequent exposure to its applications in education and research [14]. Research (*n* = 2) from Pakistan reported the postgraduate students’ opinion, using the interview-based study design and enquiring about only one AI tool, i.e., ChatGPT [15, 16]. The purpose of this study was to close these gaps by providing data on Pakistani PGHS students using a validated questionnaire. The current research utilized an analytical survey to explore PGHS students’ familiarity, attitudes, and practices regarding practical application of different AI tools during their postgraduate research. This will allow for comparison in subsequent studies and highlight the need for an AI-focused curriculum that can better prepare students for the reality of healthcare driven by AI.

## Hypothesis

The following hypotheses were developed in light of the study’s goals. It was hypothesized that PGHS students are typically familiar with AI and its applications. Furthermore, it was anticipated that students would have a largely encouraging opinion of AI and identify more advantages than disadvantages, especially in relation to efficiency, information access, and clinical decision assistance. It was predicted that a sizable portion of PGHS students will actively use AI technologies in their research, demonstrating both awareness and hands-on experience with AI. Finally, students’ attitudes, or practices with AI in research were statistically significantly correlated with their characteristics (practicing specialty, computer literacy, program, and number of publications).

## Methodology

### Aim of the study

The aim of this study was (1) to determine PGHS’s familiarity with AI (2), to explore how PGHS students view AI in terms of its advantages and disadvantages, and (3) to assess the practices of PGHS regarding the use of AI.

### Study design and duration

The study employed a cross-sectional, analytical survey design to assess PGHS’s familiarity, attitudes, and practice about the use of artificial intelligence (AI) in research. The current study was conducted for the period of two months (from 1st May to 30th June 2025).

### Sample size and study population

The study population comprised PGHS students enrolled in different postgraduate programs at universities in Karachi, including medicine, dentistry, pharmacy, nursing, and physiotherapy. Their enrollment was verified through official lists provided by the respective university administrations. The sample size of at least 246 participants was calculated using online software OpenEpi^®^, based on the prevalence of positive attitude towards AI as 80% [17], with 95% a confidence interval, 80% power of test, and 5% margin of error.

### Inclusion/exclusion criteria

PGHS students enrolled in postgraduate programs at two public and two private universities in the metropolitan city of Karachi were included in the current study. Age was above 22 years, and both genders were included. Students were eligible to participate if they provided informed consent to contribute to the study. The students who did not give answers to all items were excluded.

### Data collection procedure

The non-probability convenient sampling technique was used for the study. The directors and deans of postgraduate program approached and asked them to distribute the link of the Google form to their students currently enrolled in different postgraduate programs via the students’ email IDs. Authors briefed respondents about the rationale of the study in the form of a descriptive paragraph incorporated at the start of the questionnaire.

### Questionnaire development

A structured questionnaire was adopted from previously validated instruments [18, 19] and minimally adapted to reflect the PGHS context. Permissions were taken from the corresponding authors of those studies. The questionnaire’s items were thoroughly examined to assure clear and unambiguous phrasing during the questionnaire development process and were modified to remove any potential bias. Additionally, the scale’s suitability and alignment with the item wording were confirmed. For this purpose, a panel comprising two physicians, two dentists, two pharmacists, and two nurses reviewed the adapted questionnaire. These experts (*n* = 08) were asked to assess the relevance and importance of each item in the questionnaire. In addition, they were encouraged to revise existing items and propose new ones to enhance the questionnaire’s applicability to the current scenario.

Following their feedback, five items were removed due to irrelevance or redundancy, resulting in a refined 26-item version of the questionnaire excluding *n* = 09 items of demographics of respondents. The final draft was structured into four sections. Section A comprised demographics of respondents (*n* = 09). Section B included questions about PGHS students’ familiarity with AI (*n* = 04). Section C was comprised of questions to have students’ attitude toward AI (*n* = 16). Section D was about their practice (*n* = 06). Section C and D items were evaluated using a five-point Likert scale ranging from strongly agree to strongly disagree. For these items, “Strongly agree” and “Agree” responses were categorized as reflecting a positive opinion. Additionally, the items in section B were designed with dichotomous (binary) response options, i.e., YES or NO.

A pre-test was conducted to ensure that the scale offered sufficient variability and captured behavioral intentions, making it a practical tool for understanding students’ feedback. Twenty students participated in the pre-test to evaluate the questionnaire’s readability and applicability, and no feedback suggested that any changes were necessary. The test-retest method was used to measure reliability. A week separated the two administrations of the questionnaire, which was completed by the same 20 students on Saturday and Saturday of the following week. The total scores from both tests were then correlated, yielding an intra-class correlation value of 0.924, indicating excellent reliability. Exploratory factor analysis was used to assess the construct validity of the attitude and practice scales in the questionnaire. It was designed electronically using Google Forms, and data were collected by distributing the survey link to the postgraduate students. Furthermore, Confirmatory Factory Analysis (CFA) was also performed. The CFA results provided insight into the factor structure and helped evaluate the reliability and validity of the measurement instruments. Specifically, CFA allows to test the hypothesized relationships between the observed variables and the underlying latent constructs, providing a rigorous evaluation of the measurement model.

### Ethical consideration

Participation in the study was anonymous and voluntary. Students who declined to participate were respectfully excluded from the study. Informed consent from each participant was obtained. Participants were not forced to participate if they were not willing. They were briefed about the purpose of the study, and they were assured that the identification of the participants would not be revealed and would be kept anonymous. The Google form included a consent section. Only after participants clicked the “I give consent” option did the form moved forward. The study was approved by the Ethical Review Board, Sohail University (Protocol ID: 00110/25, dated April 25th 2025).

### Statistical analysis

Data was downloaded as an excel sheet from Google Forms and analyzed using SPSS version 27.0 of the Statistical Package for Social Sciences (SPSS) and R Studio software. Descriptive statistics were presented with the help of frequencies and percentages for all items in the questionnaire. Non-parametric Chi-square / Fisher Exact test was used to find any significant associations between the items and students’ characteristics (practicing specialty, computer literacy, program, and number of publications). A *p*-value ≤ 0.05 was considered significant. The nature of attitude variables is categorical and allows the use of non-parametric test.

## Results

### Construct validity

The construct validity of the attitude and practice scales in the questionnaire was tested using exploratory factor analysis. The attitude domain’s final item count was 16. Factor analysis was conducted on students’ attitude scale, revealing a single common factor with no weak loadings (all items ≥ 0.40). Factor loading ranged from 0.402 to 0.675, explaining 34.31% of the variation in the data (Table 1). The scale demonstrated acceptable reliability, with a Cronbach’s alpha of 0.866. Factor analysis of the students’ practice scale (6 items) revealed a single common factor with all loadings exceeding 0.40 (Table 2). Its factor loading ranged from 0.571 to 0.824 and explained 51.86% of the variance in the data. The scale demonstrated acceptable reliability with a Cronbach’s alpha of 0.811.

**Table 1:** Factor analysis of the “students’ attitude” scale

Items	Common Factor = 34.31% of variance loadings
1	0.402
2	0.408
3	0.629
4	0.675
5	0.518
6	0.542
7	0.590
8	0.634
9	0.632
10	0.654
11	0.492
12	0.626
13	0.614
14	0.637
15	0.597
16	0.633
Eigen value 5.490	

**Table 2:** Factor analysis of the “students’ practice” scale

Items	Common Factor = 34.31% of variance loadings
1	0.634
2	0.617
3	0.824
4	0.816
5	0.810
6	0.571
Eigen value 3.112	

### Confirmatory factor analysis (CFA)

#### Student attitude

##### Chi-square test

The significant *p*-value (0.070) indicates that the observed and predicted covariance matrices did not differ significantly, suggesting a good fit. CFI (Comparative Fit Index) and TLI (Tucker-Lewis Index) values are 0.986 and 0.915 respectively (> 0.9 recommended), indicating a reasonably good fit. RMSEA (Root Mean Square Error of Approximation) value is 0.051 (< 0.06 acceptable) whereas SRMR (Standardized Root Mean Square Residual) values is 0.069 (< 0.08 acceptable) indicating an acceptable fit.

#### Student practice

##### Chi-square test

The significant *p*-value (0.052) indicates that the observed and predicted covariance matrices did not differ significantly, suggesting a good fit. CFI and TLI values are 0.979 and 0.989 respectively (> 0.9 recommended), indicating a reasonably good fit. RMSEA value is 0.042 (< 0.06 acceptable) whereas SRMR values is 0.078 (< 0.08 acceptable) indicating an acceptable fit.

### PGHS students’ characteristics

A total of 257 PGHS students (257/300, response rate = 85.66%) from different universities of Karachi (*n* = 04) participated in the study and responded to the questionnaires. The sample was predominantly male (72.4%) with female participants representing 27.6%. Most participants (73.5%) were young professionals aged 20–35 years (Table 3). Master’s students formed the largest group (68.1%), PhD candidates represented 24.1% and FCPS (Fellowship) participants were the smallest group at 7.8%. It is clear that pharmacists were the largest professional group (38.1%), followed by physicians (26.8%) and nurses (16.3%). The overwhelming majority (80.2%) were university affiliated; only small percentages came from government (3.9%) or NGO (non-governmental organization) (8.6%) settings. It was observed that 63.4% reported that they are currently practicing in their specialty. Over half (52.1%) reported advanced computer literacy. The majority of the students (77%) had 0–5 publications, and only 11.7% had more than 11 publications. Researcher self-identification was evenly split between beginner (43.2%) and mid-level (43.2%), with few senior researchers (13.6%).

**Table 3:** Characteristics of the study population and their familiarity with AI (*n* = 257)

General characteristics		*N* (%)*
Gender	Male	186(72.4)
	Female	71(27.6)
Age (years)	20–35	189(73.5)
	36–51	47(18.3)
	52–67	21(8.2)
Program	Masters	175(68.1)
	FCPS	20(7.8)
	PhD	62(24.1)
Specialty	Medicine	69(26.8)
	Dentistry	27(10.5)
	Pharmacist	98(38.1)
	Nursing	42(16.3)
	Physiotherapist	21(8.2)
Affiliation	Gov. Organization	10(3.9)
	Non-Gov. Organization	22(8.6)
	Tertiary care hospital	19(7.4)
	University	206(80.2)
Practicing specialty	No	94(36.6)
	Yes	163(63.4)
Computer literacy	Basic	92(35.8)
	Intermediate	31(12.1)
	Advanced	134(52.1)
Number of publications	0–5	198(77)
	6–11	29(11.3)
	> 11	30(11.7)
Researcher level	Beginner	111(43.2)
	Mid	111(43.2)
	Senior	35(13.6)
Familiarity with AI		
Have you ever received training of using AI tools	No	164(63.8)
	Yes	93(36.2)
Have you heard of AI tools before today	No	6(2.3)
	Yes	251(97.7)
Are you aware of how artificial intelligence (AI) is affecting research?	No	50(19.5)
	Yes	207(80.5)
Have you ever conducted research using AI-powered tools?	No	69(26.8)
	Yes	188(73.5)

*N (%) represents frequency (percentage)

### PGHS students’ familiarity with AI

Table 3 shows the familiarity of PGHS students with AI. A significant majority (63.8%) reported no formal training in AI tools. Only 36.2% had received any AI training, indicating a substantial training gap. Nearly all participants (97.7%) were aware of AI tools. Most participants (80.5%) claimed familiarity with AI’s research implications. Two-thirds (73.5%) reported actively using AI-powered tools in research, whereas the remaining students had never used AI tools.

### PGHS students’ attitude toward AI

Table 4 depicts attitude towards AI among PGHS students. Distribution, frequency (%) of all 16 items are mentioned, furthermore their associations with practicing specialty, program and publication are reported with the help of chi-square or Fisher Exact Tests. It is obvious that most students agreed or strongly agreed that AI is and will be useful in academic research (93%). Almost 60% of students strongly agreed or agreed that AI will transform health care and the clinical research industry. It is observed that nearly two-thirds of the study participants (63.8%) found AI use in research ethically acceptable, though 12.5% disagreed or strongly disagreed. More than three quarters of the postgraduate students agreed that AI improves research efficiency/productivity. While responding to the item about listing AI as an author in scientific publications, nearly one quarter of the students disagreed with that. About 25% of participants disagreed that in the future AI will replace the functions of researchers. Approximately 60% of participants strongly agreed or agreed that AI-generated results are not accurate. The majority (73.9%) believed AI could replace language editors. Neutral responses were notable for AI’s role in peer review (19.5%) and AI authorship (24.5%); results generated by AI tools are not accurate (28.4%).

**Table 4:** Attitude towards AI among postgraduate health science (PGHS) students

		Frequency (%)	Frequency (%)	Frequency (%)	Frequency (%)	Frequency (%)	*P*-values			
	Items	Strongly agree	Agree	Neutral	Disagree	Strongly disagree	Practicing specialty	Computer literacy	Program	Publication
1	During the coronavirus epidemic, AI technology provided a competitive advantage in the clinical research industry by preserving social distance?	58(22.6)	93(36.2)	92(35.8)	12(4.7)	2(0.8)	**0.001** ^**F**^	**0.025** ^**F**^	**0.001** ^**F**^	0.519^F^
2	Artificial intelligence will transform health care and the clinical research industry as a whole?	55(21.4)	98(38.1)	86(33.5)	13(5.1)	5(1.9)	**0.003** ^**F**^	0.123^¶^	**0.003** ^**F**^	0.715^F^
3	I think AI tools is/ will be useful in academic research	98(38.1)	141(54.9)	15(5.8)	0(0)	3(1.2)	**< 0.001** ^**F**^	**0.002** ^**F**^	0.537^F^	0.715^F^
4	I think AI tools is/will be useful in the peer review of articles.	70(27.2)	129(50.2)	50(19.5)	7(2.7)	1(0.4)	**< 0.001** ^**F**^	0.498^F^	0.312^F^	0.082^F^
5	If AI tools help with research, I think it should be listed as an author on scientific publications	36(14)	103(40.1)	63(24.5)	46(17.9)	9(3.5)	**0.004** ^¶^	0.307^F^	**0.022** ^**F**^	NA
6	In the future, I think AI will replace the functions of language editors who edit scientific publications	53(20.6)	137(53.3)	46(17.9)	18(7)	3(1.2)	**0.024** ^¶^	0.635^F^	0.504^F^	0.410^F^
7	In the future, I think AI will replace the functions of statisticians and data analyst	44(17.1)	131(51)	48(18.7)	27(10.5)	7(2.7)	**0.001** ^¶^	0.544^F^	**0.015** ^**F**^	**0.044** ^**F**^
8	In the future, I think AI will replace the functions of researchers	35(13.6)	92(35.8)	69(26.8)	45(17.5)	16(6.2)	**< 0.001** ^¶^	0.383^¶^	0.181^F^	**0.006** ^**F**^
9	I think AI tools is/will be specifically useful for paraphrasing of paragraphs	60(23.3)	142(55.3)	31(12.1)	18(7)	6(2.3)	**0.015** ^¶^	0.399^F^	0.217^F^	0.910^F^
10	I think AI tools is / will be specifically useful to search for resources	55(21.4)	153(59.5)	26(10.1)	16(6.2)	7(2.7)	**< 0.001** ^¶^	0.426^F^	0.094^F^	0.955^F^
11	I think results generated by AI tools are not accurate	42(16.3)	109(42.4)	73(28.4)	25(9.7)	8(3.1)	0.054^¶^	**0.042** ^**F**^	**0.001** ^**F**^	**< 0.001** ^**F**^
12	I think AI tools will facilitate medical services in the future (e.g. data collection from patients	50(19.5)	142(55.3)	47(18.3)	11(4.3)	7(2.7)	0.325^F^	0.242^F^	0.055^F^	0.582^F^
13	I think using AI tools in research/writing is ethically acceptable	28(10.9)	136(52.9)	61(23.7)	22(8.6)	10(3.9)	0.473^¶^	**0.018** ^**F**^	0.311^F^	**0.038** ^**F**^
14	I think AI tools can improve the efficiency and productivity of research	55(21.4)	154(59.9)	33(12.8)	10(3.9)	5(1.9)	0.143^¶^	0.162^F^	0.435^F^	0.940^F^
15	I think AI tools needs improvement to be more useful in research	63(24.5)	138(53.7)	50(19.5)	5(1.9)	1(0.4)	**< 0.001** ^**F**^	0.423^F^	0.175^F^	**0.024** ^**F**^
16	I think AI tools improve time availability for meaningful engagement	83(32.3)	139(54.1)	24(9.3)	11(4.3)	0(0)	0.062 ^F^	0.404^F^	0.517^F^	0.987^F^

A *p*-value ≤ 0.05 was considered as significant and mentioned in bold (F: fisher exact test, ¶: Chi-square test)

Practicing specialty significantly associated with 11 out of 16 items including: The role of AI in providing a competitive advantage during the coronavirus pandemic (Item 1, *p* = 0.001), Its utility in academic research (Item 3, *p* < 0.001) and the peer review process (Item 4, *p* < 0.001), AI replacing the functions of statisticians/data analysts (Item 7, *p* = 0.001) and researchers (Item 8, *p* < 0.001). This indicates that a students’ specific medical or research field is a primary determinant of their attitude towards the integration and impact of AI technologies.

Computer literacy was significantly associated with 4 of 16 items, specifically linking higher proficiency to stronger agreement with AI’s practical utility during the pandemic (Item 1, *p*-value = 0.025) and in academic research (Item 3, *p*-value = 0.002), thoughts about result generated by AI are not accurate (item 11, *p*-value = 0.042), and its ethical acceptability (Item 13, *p*-value = 0.018),

Academic program significantly influenced views on 5 of 16 items, shaping attitude about AI’s disruptive potential pandemic advantage (item 1, *p* = 0.001), transformative capacity (item 2, *p* = 0.003), formal authorship integration (item 5, *p* = 0.022), replacing roles (item 7, *p* = 0.015), and result accuracy (item 11, *p* = 0.001).

Research production significantly influenced 5 of 16 items, linking higher output to perceptions of AI as a threat to roles (replacing analysts, item 7, *p* = 0.044, item 8, researchers *p* = 0.006), result accuracy (item 11, *p* = 0.001), ethical acceptability (item 13, *p* = 0.038), and a need for improvement (item 15, *p* = 0.024). (Table 4).

### PGHS student’s practices regarding AI

**Fig. 1:**
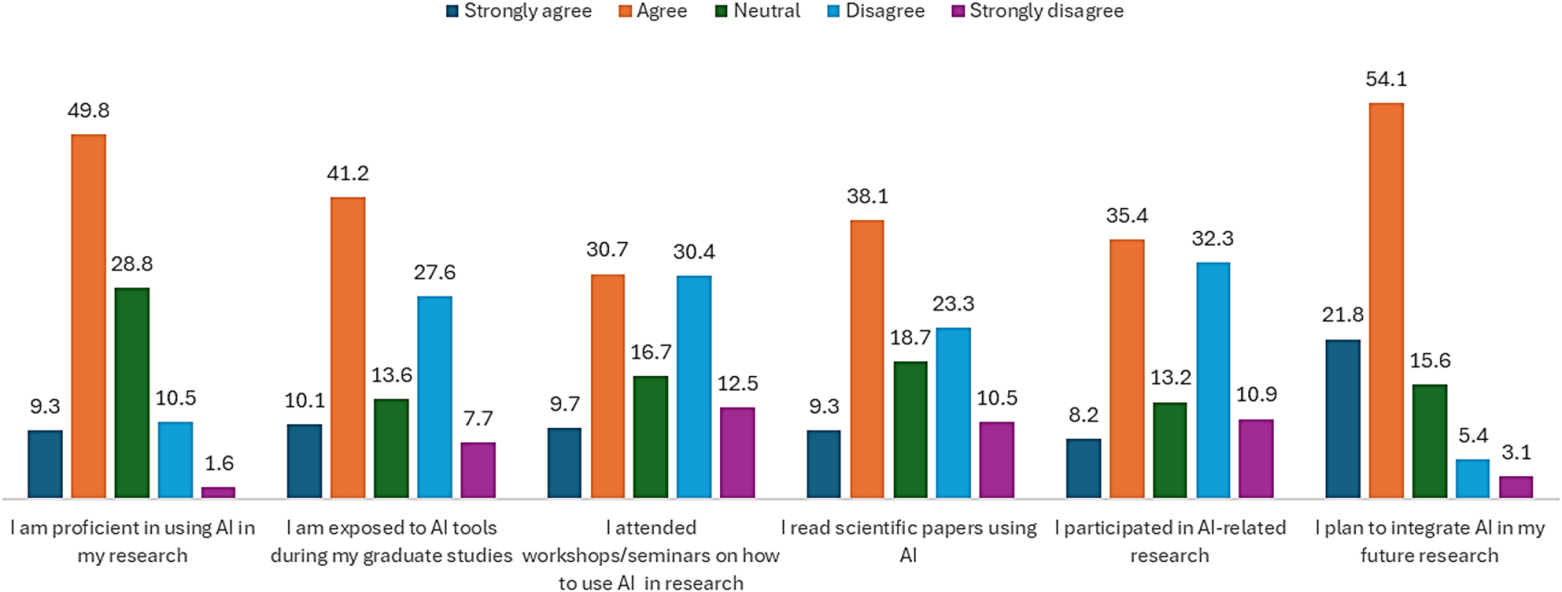
Practice of postgraduate health science (PGHS) students regarding AI

Figure 1 shows practice regarding AI among PGHS students. Half of respondents (59%) feel confident in their AI proficiency. A large neutral portion suggests others are still developing these skills. A majority experienced some AI exposure in academia, but a notable minority did not, indicating variability in educational opportunities. Attendance at AI-related training is inconsistent; roughly, a third attended, and many are neutral. Many students are engaging with AI-focused literature (47.4%), but a sizable portion still is not, possibly due to unfamiliarity or lack of interest. Students split on hands-on AI research involvement, suggesting barriers like lack of access, skills, or interest. Strong forward-looking interest in AI, indicating that even if current use is limited, many see it as part of their future work (75.9%).

## Discussion

Our study explored Pakistani PGHS students’ familiarity, attitudes and practices regarding the implication of AI in the research. To the best of our knowledge, this study is the first of its type. However, a study was conducted to explore undergraduate and postgraduate students’ perceptions and experiences of AI adoption in academic writing using an interview-based study design [15]. Another study from Pakistan examined the impact, advantages, difficulties, and potential of integrating one AI tool, i.e., ChatGPT into postgraduate education [16]. By concentrating on postgraduate health sciences (PGHS) students in Pakistan, a demographic that has not gotten much attention in previous academic research on AI, this study made a unique addition. This study adopted a more thorough approach by evaluating PGHS students’ familiarity, attitudes, and usage of AI in research, whereas earlier studies have looked at the integration of particular tools like ChatGPT or general perceptions of AI among larger student groups. It also offered important insights into how future healthcare professionals will be trained for and interact with developing AI technologies in the academic and research environment.

During the current study, a questionnaire was adopted and validated to be utilized [18, 19]. The validated questionnaire has twenty-six closed-ended items covering familiarity, attitude and practices. It showed a satisfactory level of reliability and valid psychometrics. This validated questionnaire helped to collect PGHS students’ opinions for addressing the current study’s aims. Construct validity of the attitude and practice scales was confirmed through factor analysis, which also showed that each scale had a single, robust factor with acceptable item loadings. Both scales demonstrated strong internal consistency with corresponding Cronbach’s alpha scores of 0.866 and 0.811, respectively.

PGHS students were asked about their familiarity with AI tools. Approximately all of the participants knew of AI tools. The majority of respondents were aware of the research implications of AI. Two-thirds were actively employing AI tools in their study. Our results were aligned with the earlier research reported from Pakistan assessing the familiarity of students with AI tools (i.e., 75.5–87%) [17, 20]. Similarly, Saudi medical university students were also found to be familiar with ChatGPT (> 90%), the most often used program, when familiarity was assessed. Furthermore, compared to female students, male students demonstrated a significantly higher level of familiarity with the usage of AI apps [21]. Another researcher reported that postgraduate students (56.6%) were familiar with the AI tools [18].

Postgraduate Egyptian dental students responded in a cross-sectional study that they (68.4%) did not participate in any educational activities; 31.5% of respondents said they attended webinars, seminars, or courses on AI, so there is a noticeable awareness gap [22]. Our PGHS students were also shown that one-third of PGHS students did not receive any AI training, although they were utilizing AI tools during their research.

PGHS students recognized the significance of AI in healthcare in line with the earlier studies [13]. Muhammad Mustafa Habib et al. found that Pakistani healthcare professionals had a similar favorable attitude towards AI, suggesting that medical professionals and students are becoming more conscious of AI’s potential influence on healthcare [23]. Another survey conducted with university students from a Greek social sciences department found that most students had positive opinions regarding AI [24]. Postgraduate trainee doctors from the United Kingdom perceived an overall positive impact of AI technologies on their training and education (58%) [25]. Postgraduate Chinese students of clinical medicine knew the potential advantages of AI in medical education. These advantages could greatly improve the quality and effectiveness of medical education [26].

The point of view of PGHS students about the usefulness of AI tools in research was also explored. Two-thirds of them believed that AI tools are helpful for paraphrasing paragraphs and for resource searches. A study conducted by the University of Illinois, Chicago, revealed that over half of students admitted utilization of AI during their academic writing and research, underscoring its usefulness in academic pursuits [27]. Pakistani undergraduate and postgraduate students emphasized the revolutionary potential of AI tools, stressing how they may boost productivity, increase writing quality, and help people who are struggling with language obstacles. AI tools are valuable for students, as they provide improved structure, generated ideas, and corrected language, all of which expedite the writing process [15].

The incorporation of AI in higher education raises questions regarding language competency, critical thinking, the organization and applicability of AI-generated responses, and the ethical implications of AI language models such as ChatGPT in academic assignments, highlighting how higher education is changing [28]. According to research, students are using these tools more frequently for assignments and test preparation, demonstrating their value in helping them reach their academic objectives [29]. To avoid over-reliance and to preserve academic integrity, there is also a call for careful use [30].

Students’ responses regarding the ethically permissible use of AI tools and the inaccuracy of AI-generated outcomes were blended. A study on medical students’ and faculty’s perceptions of artificial intelligence (AI) reported the increasing adoption of AI among healthcare researchers but emphasized the requirement of addressing technology limitations, training of staff, and creating ethical standards for proper use of AI in health care education [31]. According to another research on postgraduate students studying public health, 84% of respondents noted possible ethical dilemmas, 77% identified social difficulties, and another 77% expressed concern about the consequences of AI integration in public health for health equity [32]. Hence, studies exploring students’ opinions raised questions regarding accuracy, privacy, and ethics, highlighting the necessity of thorough instruction and unambiguous rules [33].

According to the report of Dwivedi, et al. ChatGPT’s difficulties in academia are widely acknowledged as a result of its lack of ethical rules and considerations. As a result, it is necessary to periodically update the research and publishing ethics [34]. Researchers typically have trouble with the writing process, so they use these tools and techniques to make their work easier. Elsevier has taken the lead and released new publication ethics rules on the use of artificial intelligence (AI) and its supplementary technologies in scientific research and writing. The goal of the policy is to give all readers, writers, reviewers, editors, contributors, etc. direction and transparency [35]. Therefore, it is necessary to create regulations addressing the ethical and privacy issues around the use of AI tools in research and academia [36].

Statistical analysis revealed that PGHS students’ opinions on AI vary greatly based on their computer literacy, specialty, number of publications, and academic program. These factors influence their perception of AI’s accuracy and potential to displace researchers and statisticians. Two factors—habit and hedonism—significantly influence postgraduate students’ adoption of AI, according to a study that examined the opinions of Malaysian postgraduate students [37]. According to a European study, medical, nursing, pharmacy, and midwifery students had a higher inclination towards the implications of AI than other healthcare students (*p* < 0.001) [38]. The opinions of tech-savvy and non-tech-savvy Pakistani students differed significantly, with the latter group showing greater dread of artificial intelligence [20]. Another study from Pakistan reported that undergraduate students’ percentage of positive attitudes varies statistically significantly depending on their academic year (*p*-value = 0.024) and medical specialty (*p*-value = 0.039) [39].

The current study also revealed that postgraduate programs do not adequately prepare students to interact with AI tools (Fig. 1). A previous report revealed that 76% of public health students supported the introduction of AI capabilities at the undergraduate level, and almost 52% of them believed they lacked the necessary training to collaborate with AI technologies [32]. Studies have also shown that students want more structured AI education to improve their preparedness for the changing labor market [40]. In order to prepare aspiring healthcare researchers for the future, integration of AI in health education and research is crucial [41]. These findings point to the necessity of formalizing and improving the current informal learning process. Students will probably be exposed to more AI applications in healthcare settings as they advance to postgraduate degrees. Therefore, a purposeful continuity of formal AI education from undergraduate to postgraduate years is a prerequisite.

Core AI competencies, including comprehending AI applications in public health, clinical decision support, and diagnostics, should covered in postgraduate healthcare curriculum. Students should be trained in the domain of health data literacy, ethical AI use, and critical evaluation of AI tools with a focus on patient safety, justice, and transparency. To train healthcare researchers for ethical and successful AI integration, interdisciplinary collaboration and hands-on experience with AI-powered systems should be promoted [42].

Importantly, this research also highlights the necessity of conducting research in order to guide the development of evidence-based teaching methods that complement and improve the progression of AI exposure in health science education [43]. Additionally, stakeholders (Higher Education Commission, Government of Pakistan, Pakistan Medical & Dental Council, Pakistan Pharmacy Council, Pakistan Nursing and Midwifery Council, etc.) would get important insights from these findings about the future application of AI in postgraduate education. They can improve the use of AI technology in postgraduate education by strategically planning and comprehending the major aspects impacting AI adoption [37].

## Limitations

The first limitation of our study is that the characteristics of our sample population were not proportionate. Males and master’s students were predominant. Additionally, younger students are over-represented. Therefore, even though our results are similar to some previous results from Pakistan, care should be taken when extrapolating them to the broader population. The size of our sample is the subject of the second constraint. The third limitation relates to the fact that data was self-reported rather than measured, which could have resulted in response bias. The study used a non-probability sampling technique, mostly because of practical limitations including the inability to obtain a complete sample frame of all Pakistani PGHS students. Selection bias could impact the sample’s representativeness due to the absence of random selection, hence it should be taken into account when interpreting the findings.

## Conclusion and recommendations

PGHS students have a positive opinion of artificial intelligence (AI) and acknowledge its potential to improve problem solving and research. However, there are serious worries about the ethical ramifications of AI, accuracy, and employment displacement, especially among students. The study emphasizes the necessity to address the worries and apprehensions of PGHS students regarding AI integration in Pakistan. To promote the broader use of AI-enabled healthcare, a multifaceted strategy that addresses particular issues, provides information, and provides tailored training is required. The implication of AI in the healthcare sector necessitates improving PGHS student perceptions of AI by incorporating AI into the curriculum and providing ongoing training for them through workshops and seminars on AI applications and usage.

## Supplementary Information

Supplementary Material 1.

Supplementary Material 2.

## Data Availability

Data is provided within the manuscript or supplementary information files.

## Abbreviations

Artificial intelligence: artificial intelligence
PGHS: postgraduate health science students
