# Supplementary material for: Familiarity, attitude and practice of postgraduate health science students of Pakistan regarding the implication of artificial intelligence in research: an analytical survey
Source: BMC Med Educ. 2026 Jan 30;26:343. doi: 10.1186/s12909-026-08632-x (PMC12931079; doi:10.1186/s12909-026-08632-x)
Supplement: Supplementary file 1 — Supplementary Material 1. [file 12909_2026_8632_MOESM1_ESM.docx]

| **General characteristics** | |
| --- | --- |
| Gender | Male |
|  | Female |
| Age (years) | 20-35 |
|  | 36-51 |
|  | 52-67 |
| Program | Masters |
|  | FCPS |
|  | PhD |
| Specialty | Medicine |
|  | Dentistry |
|  | Pharmacist |
|  | Nursing |
|  | Physiotherapist |
| Affiliation | Gov. Organization |
|  | Non-Gov. Organization |
|  | Tertiary care hospital |
|  | University |
| Practicing specialty | No |
|  | Yes |
| Computer literacy | Basic |
|  | Intermediate |
|  | Advanced |
| Publication | 0--5 |
|  | 6--11 |
|  | >11 |
| Researcher level | Beginner |
|  | Mid |
|  | Senior |

**Questionnaire**

**Familiarity with AI**

| **Items** | |
| --- | --- |
| Have you ever received  training of using AI tools | No |
|  | Yes |
| Have you heard of AI  tools before today | No |
|  | Yes |
| Are you aware of how artificial intelligence (AI) is affecting research? | No |
|  | Yes |
|  | Maybe |
| Have you ever conducted research using AI-powered tools? | No |
|  | Yes |
|  | Maybe |

**Students’ attitude scale**

| **Sr#** | **Items** |
| --- | --- |
| 1 | During the coronavirus epidemic, AI technology provided a competitive advantage in the clinical research industry by preserving social distance? |
| 2 | Artificial intelligence will transform health care and the clinical research industry as a whole? |
| 3 | I think AI tools is and will be useful in research. |
| 4 | I think AI tools is and will be useful in the peer review of research articles. |
| 5 | I think AI tools should be included as authors on scientific articles if they aid in research. |
| 6 | I think AI will eventually take the position of language editors who edit scientific publications. |
| 7 | I think AI will eventually take the place of statisticians and data analysts. |
| 8 | I think that in the future, AI will take the role of researchers. |
| 9 | I think AI tools are and will be especially helpful for paraphrasing paragraphs. |
| 10 | I think AI tools are and will be very helpful for resource searches. |
| 11 | I think AI-generated outcomes are inaccurate. |
| 12 | I think AI tools will make medical services easier in the future (e.g., patient data gathering). |
| 13 | I think it is ethically permissible to use AI tools for writing and research. |
| 14 | I think AI tools can increase research productivity and efficiency. |
| 15 | I think that they need to be improved for AI tools to be more beneficial in research improved. |
| 16 | I think AI tools improve time availability for meaningful engagement |

**Students’ practice scale.**

| **Sr#** | **Items** |
| --- | --- |
| 17 | I am proficient in using AI in my research |
| 18 | I am exposed to AI tools during my graduate studies |
| 19 | I attended workshops/seminars on how to use AI in research |
| 20 | I read scientific papers using AI |
| 21 | I participated in AI-related research |
| 22 | I am planning to integrate AI in my future research |
