# Supplementary figures and images for: Familiarity, attitude and practice of postgraduate health science students of Pakistan regarding the implication of artificial intelligence in research: an analytical survey
Source: BMC Med Educ. 2026 Jan 30;26:343. doi: 10.1186/s12909-026-08632-x (PMC12931079; doi:10.1186/s12909-026-08632-x)

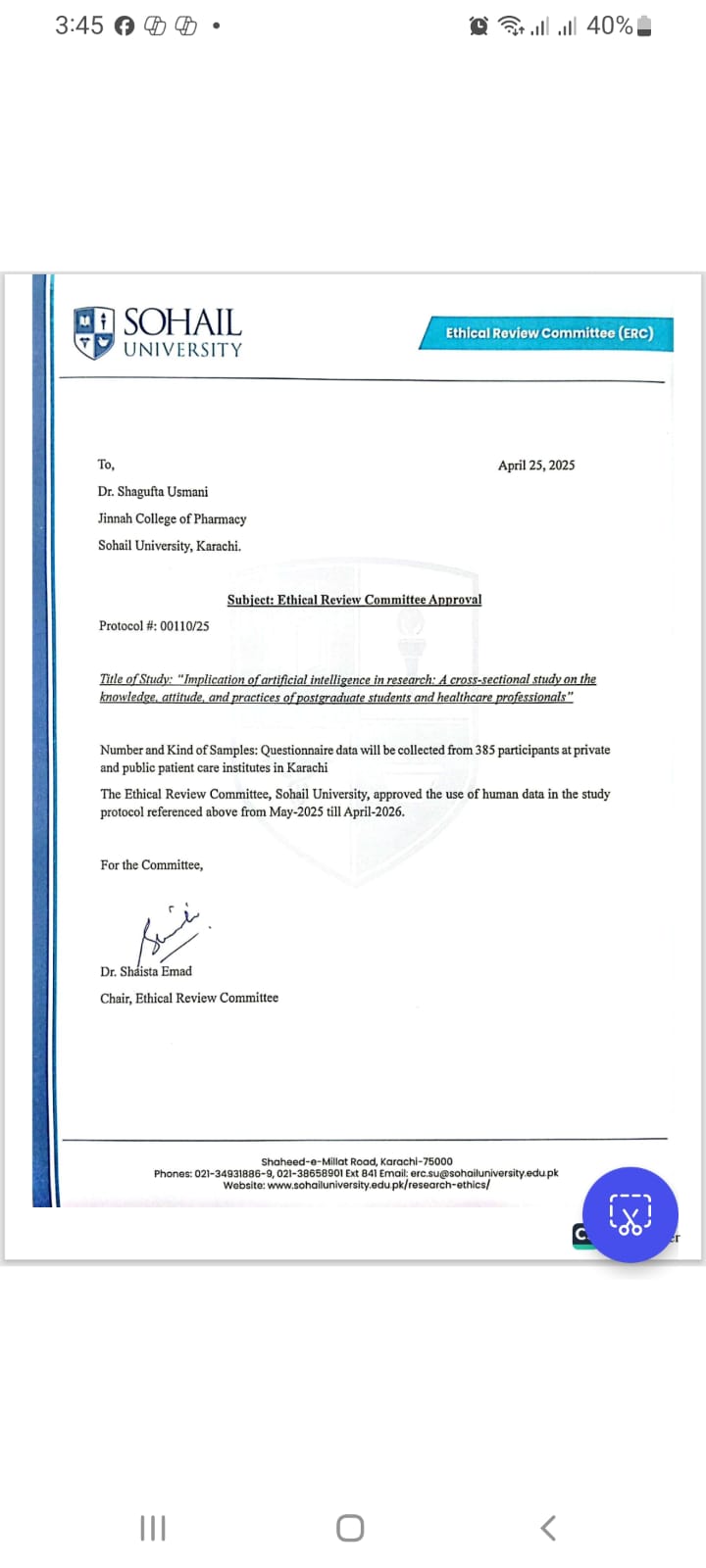

Supplement: Supplementary file 2 — Supplementary Material 2. [file 12909_2026_8632_MOESM2_ESM.jpg]
